# The SAMHD1-mediated block of LINE-1 retroelements is regulated by phosphorylation

**DOI:** 10.1186/s13100-018-0116-5

**Published:** 2018-03-28

**Authors:** Alexandra Herrmann, Sabine Wittmann, Dominique Thomas, Caitlin N. Shepard, Baek Kim, Nerea Ferreirós, Thomas Gramberg

**Affiliations:** 1 Institute of Clinical and Molecular Virology, Friedrich-Alexander University Erlangen-Nürnberg, Schlossgarten 4, 91054 Erlangen, Germany; 2 pharmazentrum frankfurt/ZAFES, Institute of Clinical Pharmacology, Goethe-University, Theodor Stern Kai 7, 60590 Frankfurt am Main, Germany; 3 Center for Drug Discovery, Department of Pediatrics, Emory Center for AIDS Research, Emory University, Children’s Healthcare of Atlanta, Atlanta, GA 30322 USA; 4 College of Pharmacy, Kyung-Hee University, Seoul, South Korea

**Keywords:** LINE-1, SAMHD1, Endogenous retroelements, Restriction factor, Aicardi-Goutières syndrome, Intrinsic immunity

## Abstract

**Background:**

The restriction factor SAMHD1 regulates intracellular nucleotide level by degrading dNTPs and blocks the replication of retroviruses and DNA viruses in non-cycling cells, like macrophages or dendritic cells. In patients, inactivating mutations in *samhd1* are associated with the autoimmune disease Aicardi-Goutières Syndrome (AGS). The accumulation of intracellular nucleic acids derived from endogenous retroelements thriving in the absence of SAMHD1 has been discussed as potential trigger of the autoimmune reaction. In vitro, SAMHD1 has been found to restrict endogenous retroelements, like LINE-1 elements (L1). The mechanism, however, by which SAMHD1 blocks endogenous retroelements, is still unclear.

**Results:**

Here, we show that SAMHD1 inhibits the replication of L1 and other endogenous retroelements in cycling cells. By applying GFP- and neomycin-based reporter assays we found that the anti-L1 activity of SAMHD1 is regulated by phosphorylation at threonine 592 (T592). Similar to the block of HIV, the cofactor binding site and the enzymatic active HD domain of SAMHD1 proofed to be essential for restriction of L1 elements. However, phosphorylation at T592 did not correlate with the dNTP hydrolase activity of SAMHD1 in cycling 293T cells suggesting an alternative mechanism of regulation. Interestingly, we found that SAMHD1 binds to ORF2 protein of L1 and that this interaction is regulated by T592 phosphorylation. Together with the finding that the block is also active in cycling cells, our results suggest that the SAMHD1-mediated inhibition of L1 is similar but not identical to HIV restriction.

**Conclusion:**

Our findings show conclusively that SAMHD1 restricts the replication of endogenous retroelements in vitro. The results suggest that SAMHD1 is important for maintaining genome integrity and support the idea of an enhanced replication of endogenous retroelements in the absence of SAMHD1 in vivo, potentially triggering autoimmune diseases like AGS. Our analysis also contributes to the better understanding of the activities of SAMHD1 in antiviral defense and nucleotide metabolism. The finding that the phosphorylation of SAMHD1 at T592 regulates its activity against retroelements but not necessarily intracellular dNTP level suggests that the dNTP hydrolase activity might not be the only function of SAMHD1 important for its antiviral activity and for controlling autoimmunity.

**Electronic supplementary material:**

The online version of this article (10.1186/s13100-018-0116-5) contains supplementary material, which is available to authorized users.

## Background

The SAM and HD domain containing protein 1 (SAMHD1) has been identified as major block to HIV-1 infection in myeloid cells and resting T cells [1–3]. SAMHD1 acts as a dNTP triphosphohydrolase and has been shown to contribute to the cell cycle-dependent regulation of intracellular dNTP levels [4–6]. In non-dividing cells, SAMHD1 is thought to limit retroviral infectivity by depleting the intracellular dNTP pool and thereby inhibiting efficient reverse transcription [7, 8]. Several groups also reported an interaction of SAMHD1 with nucleic acids, especially single strand RNA [9–11]. In addition, although controversially discussed [12, 13], SAMHD1 has been shown to contain an RNA exonuclease function and has been reported to directly degrade incoming HIV-1 genomic RNA [14, 15]. The antiviral activity of SAMHD1 is regulated by phosphorylation at threonine 592 (T592) in a cell cycle-dependent manner [16, 17]. In cycling cells, the cyclin-depended kinases (CDK) 1 and 2 in concert with cyclin A2 have been shown to phosphorylate T592 and thereby inactivate SAMHD1 [16, 18, 19]. In resting cells, however, this phosphorylation is lost and SAMHD1 is rendered antiviral active. Whether the dNTPase activity of SAMHD1 is also regulated by phosphorylation is unclear. While two initial publications showed that constitutive inactive, phosphomimetic mutants of SAMHD1 were still able to reduce intracellular dNTP levels when overexpressed in non-dividing monocytic cells, more recent in vitro studies suggest that the kinase-mediated phosphorylation at T592 might reduce the dNTP hydrolase activity of SAMHD1, at least in vitro [16, 17, 20–22].

In patients, mutations in *samhd1*, among other genes, have been associated with the rare hereditary autoimmune disease Aicardi-Goutières Syndrome (AGS) [23]. Due to the enzymatic functions of the genes involved in AGS, it has been hypothesized that aberrant nucleic acids, most likely DNA, trigger the autoimmune reaction. In the absence of SAMHD1, DNA fragments resulting from error-prone DNA repair or DNA replication, or the enhanced replication of endogenous retroelements have been discussed as potential trigger of the autoimmune reaction. Interestingly, SAMHD1 has been shown to block the retrotransposition of endogenous retroelements in cell culture [24, 25]. Long interspersed element 1 (LINE-1 or L1) is the only autonomously active retrotransposon in humans and about 17% of the genome is derived from L1 sequences [26, 27]. L1 elements are about 6 kb in length, encode three open reading frames, and lack a typical retroviral LTR promoter. While still little is known about the product of the recently discovered ORF0 open reading frame [28], which is transcribed from the L1 promoter in antisense direction, the proteins encoded by the open reading frames 1 (ORF1p) and 2 (ORF2p) are well characterized (reviewed in [29]). While ORF1p possesses an RNA binding activity and is the main component of the L1 ribonucleoprotein complex (RNP), the enzymatic activities of L1 RNPs are encoded by ORF2p, which acts as an endonuclease and a reverse transcriptase. Both functions are necessary for reverse transcribing and inserting L1 into the genome by target-primed reverse transcription (TPRT). Novel L1 retrotransposition events can destabilize genome integrity and cause disease by insertional mutagenesis, insertion of splice sites, recombination, transcriptional activation of nearby genes, or by the activation of non-autonomous short interspersed elements (SINEs), like Alu elements [30]. To this date, novel L1-mediated retrotransposition events have been identified as the disease-causing mutations in more than 120 patients, for example in cases of Duchenne muscular dystrophy or Hemophilia (reviewed in [31]). To protect from these insertions and to maintain genome integrity, the activity of L1 elements is controlled by different mechanisms, like promoter methylation, small RNA species, and by inhibitory host factors, like MOV10, APOBEC3, ZAP, or ADAR [32–38].

In addition, two studies have reported a block of L1 retrotransposition by SAMHD1 using in vitro reporter assays [24, 25]. The mechanism, however, by which SAMHD1 inhibits endogenous retroelements, remains ill described. While Zhao et al. report that SAMHD1 inhibits L1 independently of its enzymatic activities by reducing the cellular ORF2 protein levels. Hu and colleagues describe the necessity for the enzymatic activity of SAMHD1 [24, 25]. Furthermore, Hu et al. propose a dNTPase-independent mechanism of restriction and suggest that SAMHD1 induces the sequestration of L1 in cytoplasmic stress granules. Since both proposed mechanisms contradict each other and greatly differ from what is known for the SAMHD1-mediated restriction of retroviruses, we characterized the mechanism of SAMHD1 restriction of L1 in great detail. Here, we report that similar to the restriction of retroviruses the SAMHD1-mediated block to L1 depends on its enzymatic active site and is regulated by phosphorylation at T592. In addition, we found the dNTP hydrolase activity of SAMHD1 to be necessary but not sufficient to L1 retroelements. Interestingly, we observed a direct interaction of SAMHD1 with ORF2p, which is regulated by phosphorylation. Together, our data confirm previously identified functions to be important for SAMHD1-mediated L1 restriction and identify an additional novel mechanism important for the inhibition of endogenous L1 elements by SAMHD1.

## Results

### SAMHD1-mediated inhibition of endogenous retroelements is regulated by phosphorylation

**Fig. 1:**
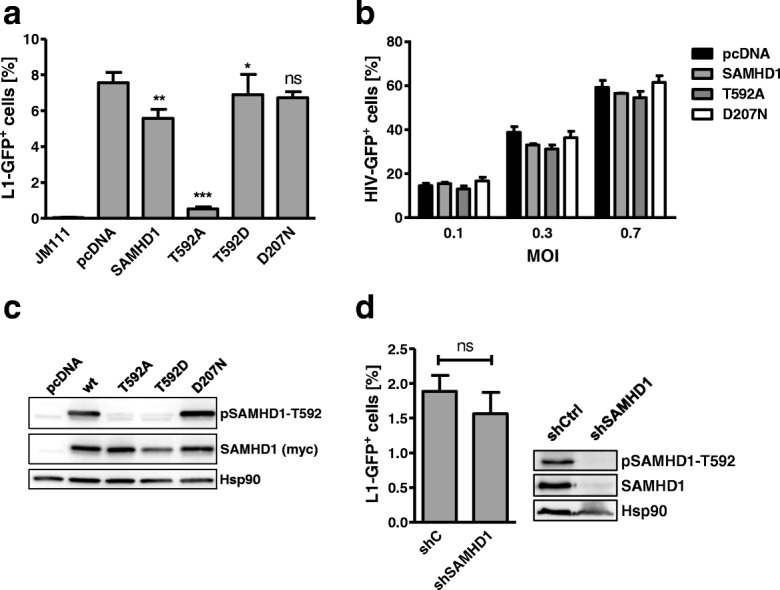
SAMHD1 T592A blocks LINE-1 but not HIV-1 replication in cycling cells. **a** 293T cells were transfected with a retrotransposition competent or a retrotransposition-defective (JM111) LINE-1 (L1)-GFP reporter plasmid and empty vector (pcDNA), SAMHD1 wt, non-phosphorylated SAMHD1 (T592A), phosphomimetic SAMHD1 (T592D), or an enzymatically inactive mutant (D207N). Five days posttransfection, GFP-positive cells were quantified by flow cytometry. The percentage of L1-GFP-positive cells is shown as average of triplicate transfections. Error bars represent the standard deviation. One out of three independent experiments is shown. Statistical analysis comparing control transfected cells with SAMHD1 expressing cells was done using one way ANOVA followed by Tukey’s multiple comparison test. * *p* < 0.05; ** *p* < 0.01; *** *p* < 0.001; ns, not significant. **b** 293T cells were transfected with empty vector (pcDNA), SAMHD1 wt, non-phosphorylated SAMHD1 T592A, or the enzymatically inactive mutant (D207N). Two days posttransfection, cells were infected with VSV-G-pseudotyped HIV-GFP reporter virus at the indicated MOIs. Three days later, GFP-positive cells were analyzed by flow cytometry. The percentage of HIV-GFP-positive cells is depicted as average of triplicate infections with error bars indicating the standard deviation. One out of three independent experiments is shown. **c** 293T cells were transfected with the indicated SAMHD1-myc expressing vectors. Lysates were analyzed 2 days posttransfection by immunoblot. Membranes were probed with phosphoT592-specific, myc-specific, and HRP-containing corresponding secondary antibodies. One out of three independent experiments is shown. **d** 293T shC or shSAMHD1 cells were transfected with L1-GFP reporter plasmid. GFP-positive cells were quantified 5 days posttransfection by flow cytometry. The percentage of L1-GFP-positive cells is shown as average of triplicate transfections. Error bars represent the standard deviation. Statistical analysis was done using an unpaired, two-tailed student’s t test. ns, not significant. The shRNA-mediated knockdown and the phosphorylation status of SAMHD1 were analyzed by immunoblot with a SAMHD1-specific and a phosphoT592-specific antibody. One out of three independent experiments is shown

To determine whether SAMHD1 influences the replication of L1 elements we employed a well-established retrotransposition reporter assay. Upon transfection of an L1-GFP reporter plasmid (99-PUR-RPS-EGFP) into 293T cells, the GFP reporter gene expression serves as a surrogate marker for successful retrotransposition [39]. The CMV-GFP cassette, which is inserted in reverse orientation in the 3’ UTR of L1 and is interrupted by an antisense intron, is expressed only when the L1 transcript is spliced, reverse transcribed, inserted into the genome, and GFP transcripts are generated. GFP-positive cells were quantified 5 days posttransfection by flow cytometry. In contrast to previous publications, we only found a weak reduction of L1-GFP activity in the presence of cotransfected 3′ myc-tagged SAMHD1 wild-type protein (wt) (Fig. 1a), with an average reduction of 40% compared to empty vector transfected control cells (Additional file 1: Figure S1). In addition, the enzymatically inactive mutant SAMHD1 D207N did not restrict L1-GFP suggesting that an enzymatically active HD domain is necessary to impede L1 retrotransposition (Fig. 1a, Additional file 1: Figure S1). The antiretroviral activity of SAMHD1 is regulated by phosphorylation of threonine at position 592 (T592), which is mediated by the cell cycle-dependent kinases CDK1 and CDK2 [16, 17]. In cycling cells, SAMHD1 is phosphorylated by CDKs at T592 and therefore thought to be inactive against HIV-1 and other exogenous retroviruses. In resting cells, like myeloid cells or resting CD4 T cells, however, T592 is not phosphorylated and SAMHD1 is antiviral active [1–3]. In line with these findings, we found transiently transfected SAMHD1 to be inactive against HIV-GFP reporter virus infection in cycling 293T cells, correlating with its phosphorylation status (Fig. 1b, c). While exogenous SAMHD1 was phosphorylated at T592 in cycling 293T cells, we could not detect phosphorylation of endogenous SAMHD1 in control cells in the western blot of Fig. 1c. This is most likely due to its low expression level resulting in a very faint band next to the strong signal of overexpressed SAMHD1. Next, we asked whether the phosphorylation status of SAMHD1 at T592 also affects its activity against L1. We therefore transiently expressed L1-GFP together with wt SAMHD1 or mutant proteins, in which we replaced the phosphorylation site T592 with alanine (T592A) or with the phosphomimetic aspartic acid (T592D), and compared the effect on L1-GFP retrotransposition. Interestingly, SAMHD1 T592A showed a strongly increased block against L1 retrotransposition (85% reduction) but not against HIV-1 infection compared to wt SAMHD1 (Fig. 1a, b). These results suggest that the anti-L1 activity of SAMHD1 is quickly regulated by phosphorylation and that only the unphosphorylated SAMHD1 mutant T592A is highly active against L1 in 293T cells. To determine whether endogenous SAMHD1 also blocks L1, we generated stable SAMHD1 knockdown cells by transducing 293T cells with a lentiviral vector encoding shRNA targeting SAMHD1 or control shRNA (shC) (Fig. 1d). Although the knockdown of SAMHD1 was very efficient, we did not observe significant differences in L1-GFP retrotransposition between shC and shSAMHD1 expressing cells (Fig. 1d). Fittingly, we found that endogenous SAMHD1 is phosphorylated in cycling 293T cells. The absence of any residual activity of phosphorylated endogenous SAMHD1, in contrast to phosphorylated exogenous SAMHD1 (Fig. 1a), might be explained by the low expression of endogenous SAMHD1 in 293T cells, which would minimize those effects. This finding supports our previous results showing that phosphorylated SAMHD1 is not active against L1-GFP (Fig. 1a). Since the SAMHD1-mediated block to L1 is phospho-T592 dependent but at the same time active in cycling cells, our results suggest a similar regulation but different mechanism of L1 inhibition by SAMHD1 compared to its block to HIV-1. The analysis also indicates that in addition to phosphorylation at T592 an additional unknown, cell cycle-dependent mechanism regulates the SAMHD1-mediated restriction of HIV-1.

**Fig. 2:**
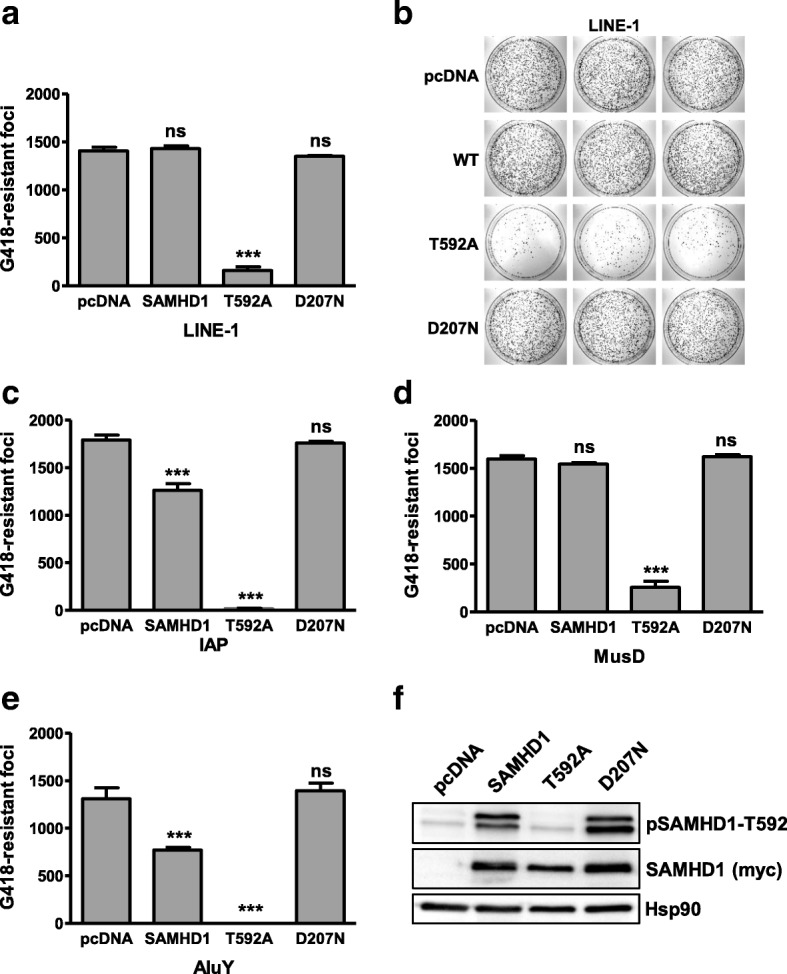
SAMHD1 T592A inhibits LINE-1, IAP, MusD, and AluY reporter elements in HeLa cells. **a** and **b** HeLa cells were transfected with a L1-neomycin reporter plasmid and either empty vector (pcDNA), SAMHD1 wt, or the indicated SAMHD1 mutants using Lipofectamine. Cells were incubated with G418-containing medium for 10 days to select for successful retrotransposition events and probed with crystal violet. **b** Crystal violet stained G418-resistant foci of L1-transfected HeLa cells. **c** HeLa cells were transfected with IAP-neomycin reporter plasmid and either empty vector (pcDNA) or the indicated SAMHD1 mutants. **d** HeLa cells were transfected with MusD-neomycin reporter plasmid and either empty vector (pcDNA) or the indicated SAMHD1 mutants. **e** HeLa HA cells were transfected with an AluY-neomycin reporter plasmid, an L1 ORF2p expression construct, and either empty vector (pcDNA), SAMHD1 wt, SAMHD1 T592A, or SAMHD1 D207N. In general, G418-resistant foci were analyzed by crystal violet staining 10 days post selection using ImageJ software. The number of resistant foci is shown as average of triplicate transfections with error bars indicating the standard deviation. Statistical analysis comparing control transfected cells and SAMHD1 expressing cells was done using one way ANOVA followed by Tukey’s multiple comparison test. *** *p* < 0.001; ns, not significant. **f** HeLa cells were transfected with the indicated SAMHD1-myc expression plasmids. Lysates were analyzed 2 days posttransfection by immunoblot. Membranes were probed with phosphoT592-specific and myc-specific primary antibodies followed by HRP-containing secondary antibodies. One of three independent experiments is shown

Next, we asked whether SAMHD1 is only active against L1 or whether other retroelements are affected as well. To exclude any cell type-specific artifacts in 293T cells, we tested the activity of SAMHD1 in HeLa cells against L1, AluY elements, and the murine LTR-containing retroelements MusD and IAP by employing well-established neomycin-based retrotransposition assays (Fig. 2) [30, 40, 41]. Therefore, we co-transfected an L1-neomycin reporter construct, in which the EGFP reporter gene was replaced by the neomycin resistance gene, with myc-tagged SAMHD1 wt, SAMHD1 T592A, SAMHD1 D207N, or empty vector. Two days posttransfection, geneticin was added to the medium and the cells were selected for successful retrotransposition events. After 14 days, resistant cell colonies were fixed, stained with crystal violet, and counted. Similar to the L1-GFP assay, we found that SAMHD1 T592A strongly inhibited L1 retrotransposition, while SAMHD1 wt was almost inactive despite being expressed to similar levels (Fig. 2a, f). Also, expression of the catalytically inactive mutant D207N did not reduce the number of resistant colonies, suggesting that the anti-L1 activity of SAMHD1 in HeLa cells is also regulated by T592 phosphorylation (SAMHD1 wt) and relies on a functional HD domain (SAMHD1 D207N). Similarly, we found that SAMHD1 T592A, and to a lesser extent SAMHD1 wt, but not SAMHD1 D207N efficiently blocked the replication of AluY elements (Fig. 2e). Modern AluY elements are active in humans and classified as SINEs. Alu elements do not encode for functional proteins and therefore hijack L1 ORF2p for successful retrotransposition. We therefore co-transfected an ORF2p expression plasmid together with expression constructs for AluY and SAMHD1. The finding that SAMHD1 T592A restricts Alu and L1 reporter elements suggests that SAMHD1 acts on L1 ORF2p or on an ORF2p-mediated activity. To determine whether the block is L1 ORF2p-specific or a more general block of endogenous retroelements, we tested the effect of SAMHD1 on the murine LTR-containing retrotransposons intracisternal A particles (IAP) and MusD (Fig. 2c, d). In neomycin-based reporter assays, we found that SAMHD1 T592A also counteracts the replication of the LTR-retrotransposons IAP and MusD, while the enzymatically inactive mutant D207N did not. Our finding that SAMHD1 represses multiple endogenous retroelements suggests a more broadly acting mechanism of restriction, like the previously described dNTP hydrolase activity or the proposed RNase activity of SAMHD1.

### The inhibition of L1 elements by SAMHD1 depends on its phosphorylation status, allosteric binding site, and functional HD domain

**Fig. 3:**
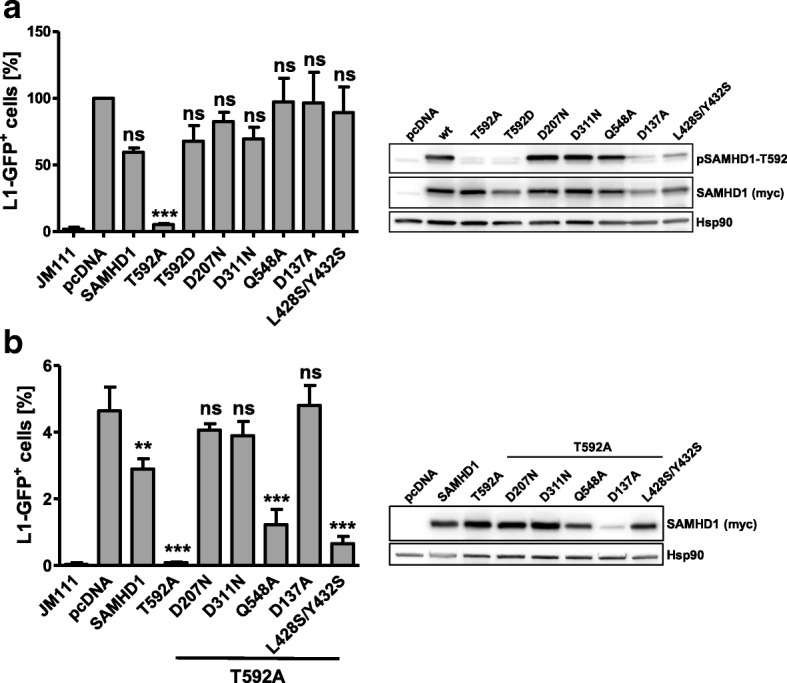
Human SAMHD1 mutants differentially inhibit LINE-1 retrotransposition. **a** 293T cells were transfected with L1-GFP reporter plasmid or the control vector JM111, together with empty vector (pcDNA), SAMHD1 wt, non-phosphorylated SAMHD1 T592A, the phosphomimetic variant SAMHD1 T592D, the HD domain mutants SAMHD1 D207N and D311N, the RNase-defective mutant Q548A, the allosteric site mutant D137A, or the oligomerization-defective mutant L428S/Y432S. The mean percentage of L1-GFP-positive cells of three independent experiments normalized on pcDNA transfected control cells is shown. Error bars represent the standard deviation of the mean. **b** 293T cells were transfected with L1-GFP reporter plasmid or control vector (JM111) and either empty vector (pcDNA), SAMHD1 wt, or the different SAMHD1 mutants shown in (**a**) but in a SAMHD1 T592A background. Five days posttransfection GFP-positive cells were quantified by flow cytometry. The percentage of L1-GFP positive cells is plotted as average of triplicate transfections. Error bars indicate the standard deviation. For western blot analysis, SAMHD1 protein expression was analyzed by immunoblot using phosphoT592-specific or myc-specific antibodies 2 days posttransfection. One out of three independent experiments is shown. For statistical analysis, one way ANOVA followed by Tukey’s multiple comparison test was used. * *p* < 0.05; ** *p* < 0.01; *** *p* < 0.001; ns, not significant

To shed light on the mechanism of L1 restriction by SAMHD1, we analyzed a variety of different human SAMHD1 mutants, which have been described in the context of HIV restriction, in retrotransposition reporter assays (Fig. 3). We introduced the corresponding mutations into wt SAMHD1 (Fig. 3a) and into the active, T592A-containing mutant (Fig. 3b). In line with our previous results (Fig. 1, Fig. 2), none of the tested proteins but the phosphorylation-defective SAMHD1 T592A significantly affected L1-GFP retrotransposition (Fig. 3a). In context of T592A, however, exchanging the aspartic acids at position 311 or 207 within the HD domain (T592A/D311N; T592A/D207N), which both cripple the enzymatic activity of SAMHD1, blocked SAMHD1 activity against L1. This finding shows that the enhanced activity of the highly active, non-regulated mutant T592A still relies on the enzymatic function mediated by an intact HD domain. In addition, interfering with the allosteric GTP-binding site of SAMHD1 (T592A/ D137A) also blocked the anti-L1 activity of SAMHD1. In contrast, the mutation Q548A, which has been reported to interfere with the proposed RNase activity of SAMHD1 [14], and the SAMHD1 multimerization mutant L248S/Y432S, had only marginal effects on the anti-L1 activity of SAMHD1. Together, the results show that, similar to the described inhibition of exogenous retroviruses by SAMHD1, the block of endogenous retroelements relies on an enzymatically functional HD domain as well as on the allosteric GTP-binding sites. In contrast, the proposed RNase activity of SAMHD1 seems not to be important for L1 restriction.

### Murine SAMHD1 blocks L1 retroelements in cell culture

**Fig. 4:**
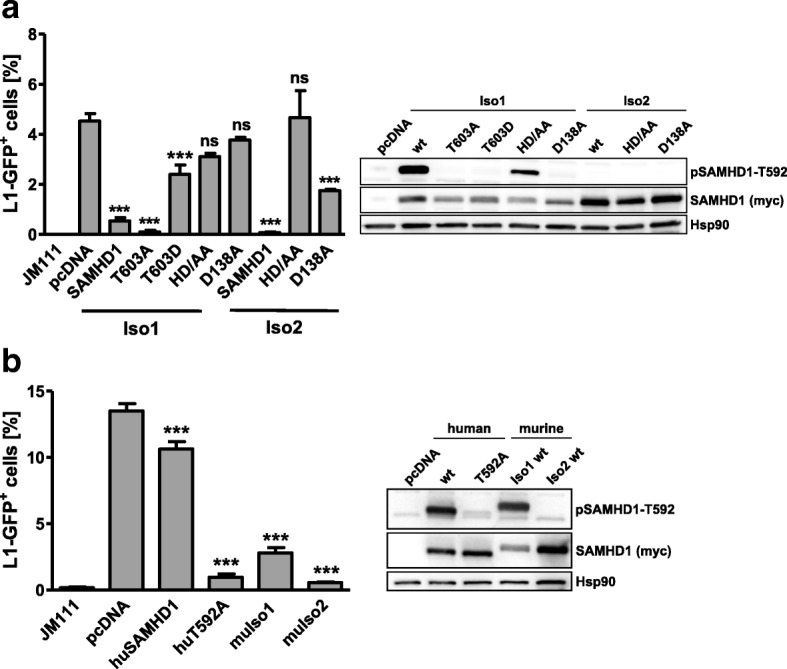
Murine SAMHD1 isoforms potently restrict L1-GFP elements. **a** 293T cells were transfected with L1-GFP reporter plasmid or control vector (JM111) together with empty vector (pcDNA), mouse SAMHD1 isoform 1 (Iso1) or isoform 2 (Iso2), non-phosphorylated mutant T603A of Iso1, phosphomimetic Iso1 mutant T603D, the HD domain mutants HD/AA, or the allosteric site mutants D138A. **b** 293T cells were transfected with L1-GFP reporter plasmid or the control vector JM111, together with empty vector (pcDNA), human SAMHD1 (huSAMHD1), huSAMHD1 T592A (huT592A), and murine SAMHD1 Iso1 (muIso1) or Iso2 (muIso2). Five days later, GFP-positive cells were determined by flow cytometry. The percentage of L1-GFP positive cells is shown as average of triplicate transfections. Error bars indicate the standard deviation. Statistical analysis comparing control transfected cells and SAMHD1 expressing cells was done using one way ANOVA followed by Tukey’s multiple comparison test. *** p < 0.001; ns, not significant. For western blot analysis, 2 days posttransfection, SAMHD1 protein expression was analyzed by immunoblot using phosphoT592-specific or myc-specific antibodies. One out of three independent experiments is shown

Two splice variants of murine SAMHD1 have been described, which differ in their carboxy-terminal domain. Interestingly, isoform 2 is missing the regulatory phosphorylation site T603, which is equivalent to T592 in human SAMHD1 [42]. We therefore compared the anti-L1 activity of the two murine isoforms and human SAMHD1 (Fig. 4). In our reporter assay, we found that both murine isoforms are able to efficiently restrict human L1 retrotransposition. Similar to human SAMHD1, mutating the enzymatically active site within the HD domain (HD/AA) or the allosteric binding site (D138A) weakens the anti-L1 activity. In line with previous publications on the antiretroviral activity of murine SAMHD1, we found that isoform 2, which is missing the regulatory phosphorylation site, is highly active against L1-GFP and that the effect of the allosteric site mutation D138A is less pronounced in isoform 2 (Fig. 4a) [43, 44]. However, due to the stronger expression of isoform 2 compared to isoform 1, the enhanced activity of isoform 2 cannot be linked directly to the missing phosphorylation site. In isoform 1, however, we found that the anti-L1 activity of the phosphorylation-mutant T603A is enhanced compared to wt protein or the phosphomimetic mutant T603D indicating that murine SAMHD1 isoform 1 is also regulated by phosphorylation (Fig. 4a). Interestingly, the phosphorylated mouse protein also seemed to be more active than human SAMHD1 (Fig. 4b). This finding might be explained by species-specific factors important for the regulation of SAMHD1, which might not interact with murine SAMHD1 in human cells. Together, we found that murine SAMHD1 also restricts L1 replication in an HD domain-dependent manner and that the regulation of the anti-L1 activity of SAMHD1 by phosphorylation most likely involves species-specific factors.

### The dNTP hydrolase activity of SAMHD1 is not regulated by phosphorylation in cycling cells

**Fig. 5:**
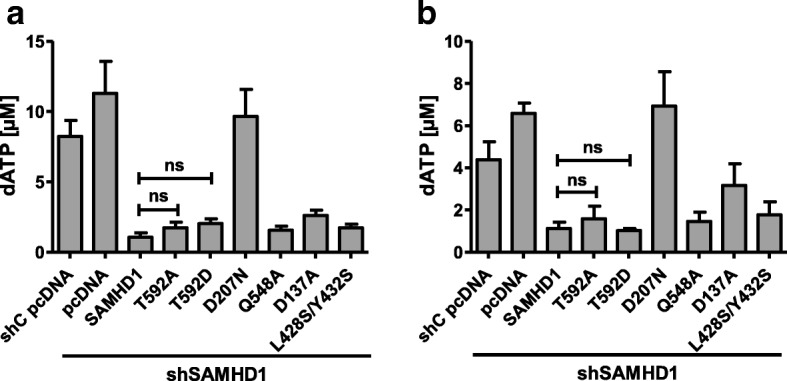
The dNTP hydrolase activity of SAMHD1 is not regulated by phosphorylation at T592 in 293T cells. 293T shC and shSAMHD1 cells were transfected with empty vector (pcDNA), SAMHD1 wt, non-phosphorylated SAMHD1 T592A, the phosphomimetic mutant T592D, the HD domain mutant D207N, the RNase mutant Q548A, the allosteric site mutant D137A, or the oligomerization-defective mutant L428S/Y432S. Two days posttransfection, cells were lysed and intracellular dNTP concentrations were determined by HPLC analysis (**a**) or single dNTP incorporation assays (**b**) The average of triplicate analysis for dATP (μM) is shown. The analysis for the other dNTPs yielded similar results. Error bars indicate the standard deviation. Statistical analysis was done using one way ANOVA followed by Tukey’s multiple comparison test. ns, not significant

Next, we asked whether the overexpression of SAMHD1 affects the intracellular dNTP level in cycling cells. We therefore transiently expressed wt SAMHD1 or the different mutants in 293T cells lacking endogenous SAMHD1 (293T shSAMHD1) and analyzed the dNTP content of the cells 48 h posttransfection by single nucleotide incorporation assay or HPLC analysis (Fig. 5a, b). Both assays were yielding very similar results. We found that the dNTP level in empty vector-transfected shSAMHD1 cells were slightly higher than in shC cells, indicating that endogenous SAMHD1 is acting as a dNTP hydrolase (only dATP levels are depicted for reasons of clarity). When we overexpressed the different SAMHD1 mutants in shSAMHD1 cells, all but the D207N mutant reduced intracellular dNTP level. Surprisingly, overexpression of the allosteric site mutant D137A in 293T also resulted in reduced dNTP level, although not as strong as wt SAMHD1. This is unexpected since recombinant SAMHD1 D137A has been shown to be defective for tetramerization and dNTPase activity in in vitro assays [45]. Interestingly, we did not find a significant difference in dNTP hydrolase activity between wt SAMHD1, T592A, and T592D suggesting that the phosphorylation at T592 regulates the anti-L1 activity of SAMHD1 but not its dNTP hydrolase activity (Fig. 5). On the other hand, the failure of SAMHD1 D207N to degrade dNTPs correlates with its inability to inhibit retrotransposition. Together, these findings suggest that a functional HD domain, and most likely its dNTPase activity, is necessary but not sufficient for L1 inhibition. Thus, our results point towards an additional, yet unknown mechanism of L1 inhibition that is regulated by phosphorylation of SAMHD1 at T592.

### SAMHD1 does not affect L1 RNA or protein expression

**Fig. 6:**
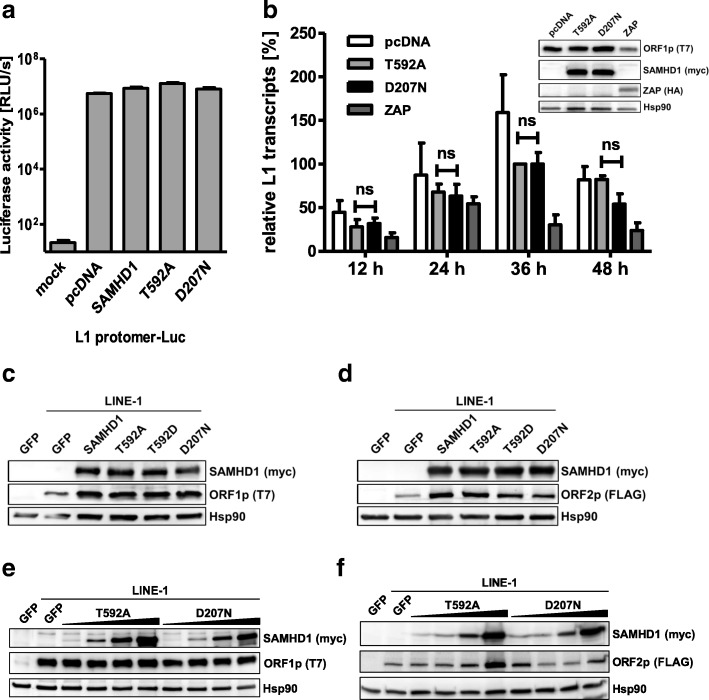
SAMHD1 T592A does not affect LINE-1 expression. **a** 293T cells were transfected with a L1 promoter construct driving the luciferase reporter gene expression (L1 promoter-Luc) together with empty vector (pcDNA), SAMHD1 wt protein, or the indicated mutants. Two days post transfection, cells were lysed and luciferase activity (relative light units, RLU) was determined in quadruplicates. The average RLU/s of three independent transfections is shown. Error bars represent the standard deviation of the mean. **b** 293T cells were transfected with the L1 reporter plasmid pAD2TE1 together with control plasmid or expression plasmids for SAMHD1 T592A, D207N, or ZAP. Total mRNA was isolated at 12, 24, 36, and 48 h posttransfection and analyzed by qRT-PCR with oligonucleotides specific for the T7 tag sequence fused to ORF1 within the transcripts of transfected L1. The average number of L1 copies per 100 ng RNA input from three independent experiments is plotted. For better comparison, all values were normalized on L1 transcripts present in T592A expressing cells 36 h posttransfection. Error bars indicate the standard deviation. Statistical analysis was done using one way ANOVA followed by Tukey’s multiple comparison test. ns, not significant. Two days posttransfection, protein expression was analyzed by immunoblot using T7- (ORF1p), myc- (SAMHD1), and HA-specific antibodies. **c** and **d** 293T cells were transfected with the L1 reporter plasmid pAD2TE1 encoding T7-tagged ORF1p or an ORF2p-3xFLAG expression plasmid together with plasmids encoding GFP, or the human SAMHD1 proteins wt SAMHD1, T592A, T592D, or D207N. Two days posttransfection, immunoblot analysis was performed to analyze SAMHD1-myc, ORF1p-T7, and ORF2p-FLAG expression levels. **e** and **f** 293T cells were transfected with the L1 reporter plasmid pAD2TE1 encoding ORF1p-T7 or ORF2-3xFLAG expression plasmid and increasing amounts of SAMHD1 T592A or D207N. To ensure a constant amount of transfected DNA, GFP expression plasmid was added to the transfection reactions. Immunoblot analysis was performed 2 days posttransfection to determine SAMHD1-myc, ORF1p-T7, or ORF2p-FLAG expression levels. One of at least three independent experiments is shown

To identify the additional mechanism or prerequisite for L1 restriction, we next determined the effect of SAMHD1 T592A expression on the different steps of the L1 life cycle (Fig. 6). First, we asked whether the promoter activity of L1 might be hampered by SAMHD1. We therefore cotransfected wt SAMHD1, SAMHD1 T592A, and SAMHD1 D207N together with a plasmid encoding the L1 promoter followed by an luciferase reporter gene into 293T cells (Fig. 6a). Forty-eight hours posttransfection we determined the luciferase activity in the cell lysates as a surrogate for L1 promoter activity. We did not detect any differences in luciferase activity between control transfected lysates (pcDNA) and the lysates of wt SAMHD1, T592A, or D207N transfected cells. Our results suggest that SAMHD1 does not inhibit L1 retrotransposition by interfering with its promoter activity. Next, we compared L1 RNA level in 293T cells expressing the active mutant SAMHD1 T592A, the inactive mutant SAMHD D207N, or the large isoform of the L1 RNA-degrading enzyme ZAP (L) [33]. Our quantitative RT-PCR approach was targeting the T7 tag sequence fused to the ORF1 open reading frame within the L1 construct pAD2TE1 to exclude the amplification of endogenous L1 RNA (Fig. 6b). In contrast to overexpression of ZAP (L), which resulted in reduced L1 RNA level 36 h and 48 h posttransfection, neither the expression of the active T592A mutant nor of inactive D207N SAMHD1 affected the L1 RNA level within transfected cells. Next, we compared L1 protein level in the presence and absence of SAMHD1 upon transient transfection in 293T cells. First, we transfected wt SAMHD1 and the SAMHD1 variants T592A, T592D, and D207N together with an L1 expression plasmid encoding T7-tagged ORF1p or a triple FLAG (3xFLAG)-tagged ORF2p expressing vector into 293T cells (Fig. 6c, d). However, we did not detect a difference in ORF1p or ORF2p protein level between the different samples. To rule out the possibility of undetected minor effects, we next transfected increasing amounts of SAMHD1 T592A or the inactive enzyme D207N together with the plasmids encoding ORF1p-T7 or ORF2p-3xFLAG into 293T cells. However, we did not detect decreased levels of ORF1p-T7 or ORF2p-3xFLAG in the presence of the highly inhibitory SAMHD1 T592A compared to the inactive mutant D207N (Fig. 6e, f).

### SAMHD1 does not impede L1 reverse transcription in vitro

**Fig. 7:**
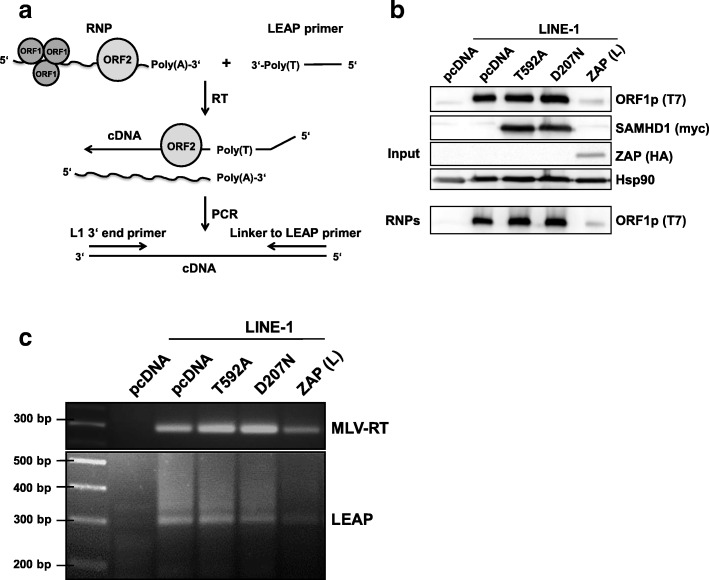
SAMHD1 does not impede ORF2p reverse transcriptase activity in LINE-1 RNPs. **a** The L1 RNP samples were purified from lysates of transfected 293T cells by ultracentrifugation through a sucrose cushion. The LEAP primer contains a linker sequence and was used to reverse transcribe L1 mRNA from RNPs. Reverse transcription occurred by either ORF2p (LEAP reaction) or exogenous MLV-RT. Synthesized cDNA was amplified by PCR with primers binding to the 3‘end of L1 cDNA and the linker sequence. **b** 293T cells were transfected with an empty vector (pcDNA) or the L1 expression vector pAD2TE1 together with expression plasmids for SAMHD1-myc T592A, SAMHD1-myc D207N, or ZAP-HA. Two days posttransfection, immunoblot membranes were probed with anti-T7 antibody (ORF1p-T7), anti-myc antibody (SAMHD1), and anti-HA antibody (ZAP). The amount of ORF1p in RNP samples after ultracentrifugation was determined by immunoblot using an anti-T7 antibody. **c** cDNA synthesized by either ORF2p or MLV-RT from L1 RNPs was analyzed by standard PCR with primers binding to the 3′ end of L1 and the linker sequence of the LEAP primer. Amplified PCR products were visualized on a 2% agarose gel

To determine how SAMHD1 affects L1 retrotransposition we analyzed the RT activity of ORF2p in vitro using a well-established RT-PCR protocol (LEAP) (Fig. 7a) [46]. We therefore transfected L1 reporter plasmid containing T7-tagged ORF1p together with pcDNA, T592A-myc, D207N-myc, or HA-tagged ZAP (L) in 293T cells. After 48 h, transfected cells were lysed and controlled for efficient protein expression by immunoblot (Fig. 7b). L1 ribonucleoprotein particles (RNPs) containing L1 genomic RNA were purified by ultracentrifugation of the lysates through a sucrose cushion and analyzed by immunoblot for ORF1p content (Fig. 7b). We could detect ORF1p in all samples but from the lysate containing the L1 restriction factor ZAP [32, 33]. Next, we generated L1 cDNA in vitro by adding dNTPs and the 3’-LEAP oligonucleotide to the purified RNPs or by additionally adding MLV reverse transcriptase to purified L1 RNA. Subsequently, we amplified the cDNA generated by ORF2p or MLV-RT by PCR using L1 and LEAP specific-primers. However, we did not find a difference in amplification of cDNA from SAMHD1 T592A and D207N samples generated by MLV-RT (Fig. 7c), indicating that SAMHD1 is not affecting the RNA content of L1 RNPs. In addition, we could not detect a difference in efficacy of the LEAP reactions based on the different RNPs generated from pcDNA, SAMHD1 T592A, or D207N containing cells. This suggests that SAMHD1 is neither degrading L1 RNA from RNPs nor is it inhibiting ORF2p-mediated RT, at least in vitro and in the presence of excess nucleotides.

### SAMHD1 directly interacts with functional L1 RNP complexes

**Fig. 8:**
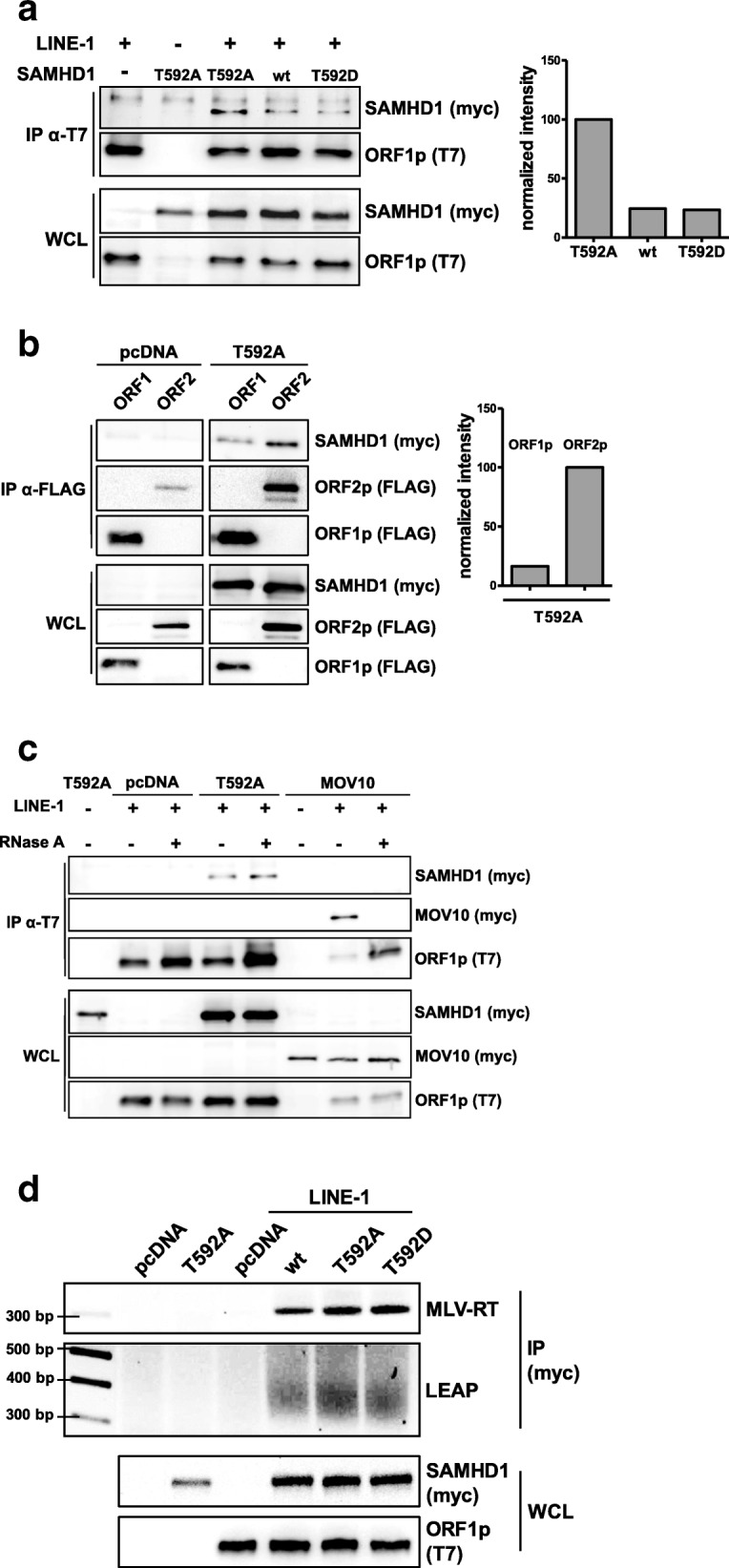
SAMHD1 interacts with LINE-1. **a** 293T cells were transfected with the L1 expression vector pAD2TE1 together with plasmids encoding wt SAMHD1, SAMHD1 T592A, or SAMHD1 T592D. Two days posttransfection, cells were lysed and ORF1p-T7 was precipitated using anti-T7 antibody coupled to magnetic beads. The precipitates were analyzed by immunoblot. SAMHD1-myc, T592A-myc, and T592D-myc signals were quantified using AIDA Image Analyzer software and normalized on the T7 signal in IP (bait) and the myc signal in WCL (input). **b** 293T cells were transfected with expression plasmids for L1 ORF1p-FLAG or ORF2p-3xFLAG together with empty vector (pcDNA) or SAMHD1 T592A. Two days posttransfection, cells were lysed and L1 proteins were precipitated with anti-FLAG antibody coupled to magnetic beads. A representative quantification of the precipitated SAMHD1 T592A signal is shown. HRP signals were quantified with AIDA Image analyzer software and normalized on bait signal and WCL input signal. **c** 293T cells were transfected with a L1 expression plasmid together with empty vector (pcDNA), SAMHD1 T592A, or MOV10. Cells were lysed 2 days posttransfection. Lysates were treated with either RNaseOUT or 15 μg/ml RNaseA prior to ORF1p-T7 precipitation using anti-T7 antibody coupled to magnetic beads. **d** 293T cells were transfected with empty vector (pcDNA), L1 expression vector and expression plasmids for SAMHD1 wt, T592A, or T592D. Cells were lysed 2 days posttransfection and SAMHD1 was precipitated with an anti-myc antibody coupled to magnetic beads. Subsequently, MLV-RT and LEAP-RT reactions were performed and amplification products were visualized on a 2% agarose gel. Input protein content was controlled by immunoblot. One out of three independent experiments is shown. WCL: Whole cell lysate, IP: immunoprecipitation

Next, we asked whether SAMHD1 directly interacts with L1 RNPs. We therefore cotransfected empty vector, myc-tagged SAMHD1 wt, T592A, or D207N mutants together with an L1 expression construct harboring a T7-tagged ORF1p into 293T cells (Fig. 8a). Upon precipitation of L1 ORF1p-T7 with T7-mAb coupled to magnetic beads, we found that all SAMHD1-myc proteins coprecipitated with L1 ORF1p (Fig. 8a). Interestingly, coprecipitation of L1 (ORF1p-T7) and the constitutively active, phosphorylation-deficient mutant T592A resulted in a more intense SAMHD1 band than precipitation of the phosphorylated proteins SAMHD1 wt and T592D (normalized densitometric intensities: T592A 100%, WT 25%, T592D 24%). This finding suggests that phosphorylation of SAMHD1 at T592 might regulate the direct interaction of SAMHD1 with L1 RNPs. To determine whether SAMHD1 interacts with ORF1p or ORF2p, we expressed SAMHD1 T592A or empty vector together with plasmids encoding either ORF1p-FLAG or ORF2p-3xFLAG (Fig. 8b). Although, we found that SAMHD1 T592A coprecipitated with both constructs, precipitation of ORF2p resulted in the strongest signal despite being expressed to the lowest level (Fig. 8b, inlet). This suggests that SAMHD1 most likely interacts with ORF2p rather than ORF1p. Although we cannot completely rule out direct binding of SAMHD1 to ORF1p, it is conceivable that the weak SAMHD1 signal upon precipitation of ORF1p might be due to the interaction of ORF1p with endogenously expressed ORF2p binding to SAMHD1. Since SAMHD1 has also been reported to bind to RNA, we next asked whether SAMHD1 directly binds to ORF2p or whether the interaction is mediated by L1 RNA. We therefore precipitated L1 RNPs via ORF1p-T7 and analyzed the binding to SAMHD1 T592A in the presence and absence of RNaseA. When we treated the cell lysates with RNaseA prior to the purification process, we found that the pull down of the control protein MOV10 is abrogated. This is in line with a previous publication reporting that the interaction of MOV10 with L1 RNPs is mediated by binding to L1 RNA [36]. In contrast, the precipitation of SAMHD1 T592A was not affected by RNase treatment, suggesting that SAMHD1 is not interacting directly with L1 RNA (Fig. 8c). To confirm our findings on the interaction of L1 with SAMHD1, we next asked whether SAMHD1 interacts with functional L1 RNPs. We therefore combined our immunoprecipitation protocol with the LEAP RT-PCR protocol described above (LEAP-IP). We found that L1 RNA was reverse transcribed by MLV-RT and amplified by PCR from SAMHD1-myc, T592A-myc, and T592D-myc precipitates (Fig. 8d, upper panel). In addition, L1 ORF2p RT activity was pulled down by all SAMHD1 proteins but not from empty vector transfected cells indicating that SAMHD1 is interacting with functional L1 RNPs within cells (Fig. 8d, lower panel).

Together our findings conclusively show that SAMHD1 inhibits retrotransposition of L1 and other endogenous retroelements and that this inhibition is mediated by an enzymatically active HD domain and is regulated by phosphorylation.

## Discussion

Mutations in SAMHD1 are associated with the autoimmune disease Aicardi-Goutières Syndrome (AGS) [23]. Since SAMHD1 blocks exogenous retroviruses at reverse transcription (RT), it is conceivable that the replication of endogenous retroelements, which also rely on RT, is enhanced in the absence of SAMHD1 and generates the molecular trigger leading to AGS in patients. Indeed, SAMHD1 has been shown to block L1 elements in vitro, however, the mechanisms proposed so far are unclear and partially contradict what is known for the SAMHD1-mediated restriction of HIV and other retroviruses [24, 25]. A better understanding of the role of SAMHD1 during endogenous retroelement replication is essential to determine whether SAMHD1 is important for L1 restriction beyond the cell culture model. Since the analysis of endogenous L1 replication in the face of many different SAMHD1 mutants and assay conditions is not feasible, we set up in vitro retrotransposition reporter assays to clarify the mechanism of L1 restriction by SAMHD1. Similar to the SAMHD1-mediated block to HIV, we found that a functional HD domain and intact cofactor binding sites are important for L1 restriction confirming the functionality of SAMHD1 in our in vitro test system.

Previously, it has been shown that the CDK1/2-mediated phosphorylation of SAMHD1 regulates its anti-HIV activity [16, 18, 19]. In contrast to the initial publication by Zhao et al., our results show that the SAMHD1-mediated restriction of retroelements in cycling cells is also regulated by phosphorylation at threonine 592 (Fig. 1) [24]. It is unclear, why wt SAMHD1 has been found to be highly active against L1 in 293T cells in the initial publication [24]. It might be that the cells used in the previous study were proliferating slower or were seeded at higher density resulting in more cells in G1/G0 phase harboring a dephosphorylated SAMHD1. Unfortunately, the authors did not control the phosphorylation status of SAMHD1. Interestingly, however, the authors did see a small increase in the anti-L1 activity of SAMHD1 T592A compared to wt SAMHD1 but did not comment on it, most likely due to the limited resolution of the L1-GFP assay presented in the manuscript. While endogenous SAMHD1 and overexpressed SAMHD1 wt protein were found to be phosphorylated and almost inactive, expression of the phosphorylation-deficient SAMHD1 mutant T592A blocked L1 retrotransposition efficiently. These results imply that also in vivo SAMHD1 is only active against L1 when it is not phosphorylated, most likely in non-cycling cells. It will therefore be very interesting in the future to analyze the phosphorylation status of SAMHD1 in cell types, in which L1 has been shown to be highly active in vivo, for example in germline cells, during early embryogenesis, or in neuronal precursor cells (reviewed in [47]).

In contrast to L1, we found that SAMHD1 T592A is not able to restrict HIV reporter virus infectivity in cycling 293T cells. This finding suggests that either both pathogens are inhibited by different functions of SAMHD1, or that the block to HIV is not active to its full extent in cycling cells compared to non-cycling cells, in which T592A has been shown to restrict HIV infection. Since SAMHD1 degrades intracellular dNTPs, we analyzed the dNTP level in 293T cells lacking endogenous SAMHD1 complemented with various SAMHD1 mutants. We found that overexpression of SAMHD1 T592A strongly reduced intracellular dNTP level in cycling 293T cells, however, only to a level that still supports efficient HIV RT (500–1000 nM) (Fig. 5) [48]. This finding might explain the lack of anti-HIV activity of SAMHD1 in 293T cells. Not much is known about the efficiency of the reverse transcriptase activity of ORF2p; however, initial in vitro results suggest a K_m_ of OPF2p for the different dNTPs between 0.38 μM and 0.83 μM [49]. Therefore, also L1 ORF2p might not be affected by the low dNTP level in SAMHD1-overexpressing 239 T cells. In line with these results, we found that overexpression of SAMHD1 in 293T cells reduced the intracellular dNTP pool independent of its phosphorylation status. SAMHD1 wt and the phosphomimetic variant T592D, which both are not or only minimally active against L1, reduced the amount of dNTPs to the same level as SAMHD1 T592A. These findings suggest that the block to L1 depends on the dNTPase activity of SAMHD1 but at the same time is regulated by a second, phosphorylation-dependent mechanism.

Hu and colleagues suggested that SAMHD1 promotes stress granule information and induces the sequestration of L1 RNPs through activation of the cytoplasmic stress granule pathway [25]. However, we did not detect enhanced stress granule formation upon transfection of SAMHD1 T592A with or without L1 compared to control cells (Additional file 2: Figure S2). In addition, we did not detect any differences in number or size of stress granules between SAMHD wt and T592A transfected cells suggesting that enhanced stress granule formation is also not regulated by SAMHD1-phosphorylation (data not shown).

Interestingly, we found that SAMHD1 co-precipitates with L1 RNPs upon expression in 293T cells and that SAMHD1 binds slightly more efficient to ORF2p than to ORF1p. Since SAMHD1 is mostly nuclear our data hint towards a direct interaction of SAMHD1 and ORF2p in the nucleus. Unfortunately, we and others were not able to detect L1 ORF2p in the nucleus of transfected cells by immunofluorescence, most likely due to the low expression levels of the enzyme [24, 25]. However, we found that the active SAMHD1 mutant T592A is pulled down by L1 RNPs more efficiently than wt or the inactive T592D mutant, suggesting that the phosphorylation of SAMHD1 might regulate the interaction of SAMHD1 with L1 (Fig. 8a). In addition, treating the precipitate with RNase left the interaction intact indicating that SAMHD1 targets L1 protein rather than L1 RNA (Fig. 8c). Together with the finding that an intact HD domain and cofactor binding site are necessary for restriction, our data suggest a model, in which only unphosphorylated SAMHD1 binds L1 RNPs efficiently within the nucleus and inhibits L1 elements by locally depleting the dNTP pool in close proximity to L1 through its enzymatic dNTP hydrolase activity. Unfortunately, it is rather difficult to analyze this locally-confined activity in vitro. In the LEAP assay, for example, the effect is cancelled out by adding exogenous dNTPs in excess and ORF2p-RT is active despite the presence of T592A (Fig. 7). A locally-confined depletion of dNTPs would explain the necessity for an intact dNTP hydrolase domain despite a relative small effect on total dNTPs within the cell and the absence of a difference between SAMHD1 T592A and T592D in reduction of overall dNTPs. Interestingly, this model of SAMHD1 activity would also be in line with findings showing that the phosphorylation of SAMHD1 at T592 regulates its anti-HIV activity but not its dNTPase activity [17, 50].

## Conclusion

Altogether, our data suggest that SAMHD1 indeed joins the ranks of the “guardians of the genome” and is able to block the replication of endogenous retroelements like L1. In contrast to previous publications, we found that the enzymatic activity of SAMHD1 is important for restriction and that the activity of SAMHD1 against retrotransposons is regulated by phosphorylation at T592. We also show that SAMHD1 directly interacts with L1 proteins and that this interaction is regulated by phosphorylation at T592. Our findings suggest that, in addition to its dNTPase activity, the direct interaction with L1 is important for efficient L1 restriction. With regard to the phospho-specific regulation of SAMHD1, it will be of high interest to determine in which cells SAMHD1 is keeping L1 in check and how this is connected to the role of SAMHD1 in preventing autoimmunity, cancer development, and maintaining genome integrity.

## Methods

### Cell lines and cell culture

HEK 293T (293T), HeLa, HeLa HA [51], and U2OS cells were cultured in Dulbecco’s Modified Eagle Medium (DMEM) supplemented with 10% fetal bovine serum (FBS), 100 U/ml penicillin, 100 μg/ml streptomycin, and 1 mM glutamine. 293T cells expressing shRNA targeting SAMHD1 or control shRNA were generated by lentiviral transduction. Three days postinfection, cells were selected with 2.5 μg/ml puromycin and efficient knockdown of SAMHD1 was confirmed by immunoblotting. Stable shRNA expressing cells were cultivated in DMEM / 10% FBS supplemented with 0.5 μg/ml puromycin.

### Plasmids

For exogenous expression of 3′ myc-tagged human and murine codon-optimized SAMHD1 proteins, wildtype (wt) protein or the indicated SAMHD1 mutants were cloned in the pcDNA6mycHis expression vector via *Hind*III and *Xba*I (Invitrogen). Mutations in SAMHD1 were introduced by overlapping PCR mutagenesis. The amplicons were digested with *Hind*III and *Xba*I. For expression of FLAG-tagged L1 ORF1, a 3’ FLAG tag was attached to ORF1 from the L1 subfamily RP by PCR. The amplicon was digested and ligated to pcDNA3.1 vector (Invitrogen) via *Apa*I and *Not*I. To generate the L1 reporter construct pAD2TE-O1F the T7 tag at the carboxy-terminus of ORF1 in pAD2TE1 was replaced with a FLAG tag by overlapping PCR. The resulting PCR product was cloned into pAD2TE1 via *Age*I and *Bst*Z17I. All constructs were validated by nucleotide sequencing. For the GFP-based L1 retrotransposition assay we used the retrotransposition-competent plasmid 99 PUR RPS EGFP based on L1-RP sequences and the defective control construct 99 PUR JM111 EGFP as described previously [39]. For the neomycin-based L1 retrotransposition assay, the plasmid pAD2TE1 was used, which is based on L1–3.1 and encodes a T7-tagged ORF1 and a TAP-tagged ORF2 protein [46, 52]. For the neomycin-based Alu retrotransposition assay the AluY reporter plasmid pAlutet was transfected together with the L1 ORF2 expression plasmid pAD500 encoding a 3xFLAG-tagged ORF2 protein derived from the L1–3.1 sequence as described previously [30, 52]. For the neomycin-based MusD and IAP replication assays we used pGL3-IAP92L23neoTNF and pCMVmus-6-DneoTNF containing a neomycin reporter cassette were used [40, 53]. To assess L1 promoter activity the L1RP-luc plasmid expressing the luciferase reporter gene under the control of the L1 promoter was transfected in HEK293T cells. The expression plasmid for human ZAP (pcDNA4-huZAP (L)-HA) was generated by Kerns and colleagues and acquired from Addgene [54].

### Retrotransposition assays

The L1-GFP reporter plasmids 99 PUR L1RP EGFP and 99 PUR JM111 EGFP (negative control) have been described previously [39]. Both plasmids contain a CMV-EGFP reporter cassette interrupted by an intron in the opposite transcriptional orientation within the 3’ UTR. EGFP is used as a surrogate marker for successful retrotransposition and is only expressed upon RNA splicing, reverse transcription, and integration. The L1 reporter plasmid and the indicated SAMHD1 expression vector or empty vector were transfected into 293T cells at a molecular ratio of 3:1 using calcium phosphate. Two days posttransfection, cells were selected by the addition of 2.5 μg/ml puromycin. After 3 days, GFP-positive cells were quantified by flow cytometry. For neomycin-based retrotransposition assays, reporter plasmids for L1 (pAD2TE1), IAP (pGL3-IAP92L23neoTNF), or MusD (pCMVmus-6-DneoTNF) were transfected into HeLa cells together with empty vector or the indicated SAMHD1 expression plasmid using Lipofectamine 2000 (Thermo Scientific). For the Alu retrotransposition assay, HeLa HA cells were transfected with the AluY reporter plasmid (pAlutet), an ORF2p expression vector (pAD500), and pcDNA-SAMHD1-myc or empty vector using FuGENE HD transfection reagent (Promega). Similar to the L1-GFP reporter construct, the neomycin gene is interrupted by an intron in the opposite transcriptional orientation and is only expressed after successful retrotransposition. One day posttransfection, cells were transferred into 10 cm dishes and after one or 2 days incubated with 500 μg/ml G418. After 10 days, cells were fixed with 1% paraformaldehyde in PBS and G418-resistant foci were stained with 0.1% crystal violet in 10% ethanol. Stained foci were quantified using ImageJ software (NIH).

### Virus preparation and HIV-GFP infection assay

HIV-GFP reporter virus was produced in 293T cells by cotransfection of an env-deficient reporter virus plasmid (pNL43-E-CMV-GFP) and a vesicular stomatitis virus glycoprotein expression plasmid (pVSV-G) at a mass ratio of 4:1 using the calcium phosphate transfection method. ShRNA-containing viral particles were produced by cotransfection of pVSV-G, the HIV packaging plasmid pCMVdeltaR8.9, and the lentiviral vector pLKO.1-puro encoding scrambled control shRNA or shRNA targeting SAMHD1 (target sequence: GCAGATGACTACATAGAGATT). Cell culture supernatant was harvested 48 h posttransfection, passed through 0.4 μm pore size filters and stored at − 80 °C. Reporter virus was titrated on 293T cells and infectivity was determined 72 h postinfection by flow cytometry. Viral particles encoding shRNA were quantified by p24 ELISA. In HIV-GFP infection assays, 293T cells were transfected with empty control vector or a vector encoding the indicated SAMHD1 proteins using calcium phosphate transfection. One day posttransfection, 1 × 10^5^ transfected cells were seeded in 6-wells and infected with VSV-G-pseudotyped HIV-GFP reporter virus at a MOI of 0.1, 0.3, and 0.7. Three days postinfection, cells were harvested and fixed with 2% paraformaldehyde. GFP-positive cells were quantified by flow cytometry.

### Immunoblot analysis

Cells were lysed in NP-40 lysis buffer (10 mM Tris-HCl pH 7.5, 150 mM NaCl, 2 mM EDTA, 0.5% NP-40, Halt Protease Inhibitor). Lysates were quantified by Bradford assay (Carl Roth). In general, 30 μg per sample were separated by SDS-PAGE, transferred onto PVDF membranes and probed with different primary antibodies. Endogenous SAMHD1 was probed with anti-SAMHD1 (3F5) antibody (novusbio); phosphorylated T592 was detected with a pT592-SAMHD1-specific antibody (ProSci). Myc-tagged proteins were probed with an anti-myc (9B11) antibody (Cell Signaling), FLAG-tagged proteins with anti-FLAG M2 antibody (Sigma), T7-tagged proteins with anti-T7 antibodies (Novagen, Abcam), and HA-tagged proteins with an anti-HA (16B12) antibody (Biolegend). To control for equal loading of cell lysates membranes were probed with anti-HSP90 α/β antibody (Santa Cruz). Subsequently, PVDF membranes were incubated with anti-mouse or anti-rabbit HRP-labeled secondary antibodies (Cell Signaling) and visualized using HRP substrate on an Intas Advanced Fluorescence Imager (Intas).

### Quantitative RT-PCR

Total RNA of 293T cells cotransfected with the L1 expression vector pAD2TE1 and the indicated SAMHD1 plasmids (ratio 3:1) was isolated at 12 h, 24 h, 36 h, and 48 h posttransfection using the NucleoSpin RNA Kit (Macherey-Nagel) according to the supplier’s manual. Then, 1 μg of purified, DNaseI-treated RNA was reverse transcribed using an oligo-dT primer and Superscript II reverse transcriptase (Life Technologies). Quantitative PCR was performed in triplicates on an ABI Prism 7500 cycler (Applied Biosystems) using 100 ng cDNA, a forward primer recognizing ORF1 (5′-gaaggaagcgctaaacatgg), and a reverse primer binding to the T7 tag region (5′ cccatttgctgtccaccag) together with SYBR green reagent (Life Technologies). Dilutions of the L1 expression plasmid pAD2TE1 served as standard curve.

### LEAP assay

The principle of the LEAP assay has been described previously [46]. Here, 293T cells were cotransfected with the L1 expression plasmid pAD2TE1 and SAMHD1 plasmid or empty vector at a molecular ratio of 1:1 using calcium phosphate transfection. Two days posttransfection, L1 ribonucleoprotein complexes (RNPs) were isolated by ultracentrifugation (168.000 × g for 2 h at 4 °C) through a 17% sucrose cushion. Precipitates containing L1 RNPs were resuspended in nuclease-free H_2_O supplemented with Halt Protease Inhibitor and total protein concentrations were adjusted to 1.5 mg/ml. To control for successful precipitation, 30 μg of the RNP samples were analyzed by immunoblot. Next, 45 μg of the RNP sample were used for L1 RNA isolation using the NucleoSpin RNA isolation kit (Macherey-Nagel) according to the supplier’s manual. Extracted RNA was treated with DNaseI using the DNA-free™ Kit (Life Technologies) according to the supplier’s manual. Of the isolated RNA, 1 μg was reverse transcribed using a 3’-RACE primer (5’-GCGAGCACAGAATTAATAC-GACTCACTATAGGTTTTTTTTTTTTVN) and M-MLV RT (Promega). To analyze ORF2p-mediated reverse transcription in vitro, 1.5 μg of the RNP precipitate together with 3’-RACE primer were added to the RT reaction. RT products generated by ORF2p or MLV RT were amplified using Phusion DNA polymerase (Thermo Scientific) together with the 3’-RACE outer primer (5’-GCGAGCACAGAATTAATACGACT) and the 5′-L1 primer (5’-GGGTTCGAAATCGATAAGCTTGGATCCAGAC). PCR products were separated on 2% agarose gels and visualized by the QUANTUM ST5 imaging system (Peqlab).

### L1 promoter assay

293T cells were transfected with the L1 promoter luciferase reporter plasmid (L1RP-luc) together with empty vector (pcDNA6mycHis) or SAMHD1 expressing vector using calcium phosphate. Two days posttransfection, cells were lysed with Cell Culture Lysis 5× reagent (Promega) and luciferase activity was quantified using commercially available components (Promega).

### Immunofluorescence analysis

For immunofluorescence analysis adherent 293T, HeLa HA, or U2OS cells were grown on coverslips in 24-well dishes. In case of 293T cells, coverslips were pre-coated with poly-lysine (Millipore). 293T cells were transfected using Lipofectamine 2000 (Thermo Scientific); HeLa HA and U2OS cells were transfected using FuGENE HD (Promega). Cells were transfected with pAD2TE-O1F together with empty vector or pcDNA6mycHis-SAMHD1 (molecular ratio 1:1). Two days posttransfection, cells were fixed with 4% paraformaldehyde and permeabilized using 0.4% saponin in PBS for 20 min at 4 °C. Antibodies were diluted in PBS containing 0.4% saponin and 1% FBS. SAMHD1-myc was detected with using a myc-Alexa488 antibody (1:250 dilution), ORF1p was probed with anti-FLAG M2 antibody (1:500 dilution) (Sigma) and a corresponding anti-mouse Alexa488 secondary antibody (Cell Signaling). Endogenous G3BP1 was probed with a primary anti-G3BP1 antibody (1:1000 dilution) (Proteintech) and a corresponding anti-rabbit Alexa647 secondary antibody (Cell Signaling).

### Immunoprecipitation assays

293T cells were lysed 48 h posttransfection in 160 mM NaCl, 50 mM Tris-HCl (pH 7.5), 1 mM EDTA, and 0.25% NP-40, supplemented with Halt Protease Inhibitor, 1 mM PMSF (Sigma), and RNaseOUT (Life Technologies). RNase inhibitors were omitted from samples treated with 15 μg/ml RNaseA (Life Technologies). FLAG- or T7-tagged L1 proteins were immunoprecipitated with anti-FLAG M2 (Sigma) or anti-T7 (Novagen) monoclonal antibodies coupled to magnetic beads (Dynabeads, Thermo Fisher). Precipitated proteins were separated by SDS page and transferred onto PVDF membranes and probed with anti-myc (9B11) antibody (Cell Signaling) anti-FLAG M2 (Sigma), or anti-T7 antibody (Novagen). Next, membranes were incubated with anti-mouse HRP light chain-specific or anti-rabbit HRP conformation-specific secondary antibodies (Cell Signaling). For immunoprecipitation of SAMHD1 combined with subsequent LEAP reaction (LEAP-IP), 293T cells transfected with pAD2TE1 and SAMHD1-myc or empty vector were lysed and myc-tagged SAMHD1 was precipitated using anti-myc antibody bound to magnetic beads (Cell Signaling). The beads harboring SAMHD1 and L1 RNPs were eluted in MLV-RT buffer (Promega). Samples for LEAP reaction were directly subjected to reaction, samples for MLV-RT reactions were incubated for 10 min at 95 °C. LEAP and MLV-RT reactions were performed as described above.

### Intracellular dNTP quantification

Intracellular dNTP levels were quantified by two different approaches, liquid chromatography-tandem mass spectrometry analysis and dNTP incorporation assay. In both cases, 293T cells expressing shRNA targeting SAMHD1 were transfected with the different SAMHD1 mutants using the calcium phosphate method. Quantification of dNTPs by liquid chromatography-tandem mass spectrometry has been described previously [42, 55]. Here, 1 × 10^6^ cells were pelleted, lysed and the analytes were extracted by protein precipitation. Samples were chromatographically separated using an anion exchange HPLC column and analyzed in a 500 QTrap mass spectrometer. The calibration ranges in the injected solution were 4–1000 ng/ml for dTTP and dCTP, 2–500 ng/ml for dATP, and 4–500 ng/ml for dGTP. Quantification by the dNTP incorporation assay was performed as described previously [8, 56]. Briefly, the lysates of 2 × 10^6^ transfected cells were incubated with 5’ P32-labeled 23-mer oligonucleotides annealed to one of four distinct 24-mer templates with a single nucleotide overhang (A, C, G, or T). The template/primer was incubated with extracted cellular dNTPs and purified HIV-1 RT. Reactions were resolved by 20% Urea-PAGE and single nucleotide incorporation was quantified by analyzing P32-containing oligomers.

## Additional files

Additional file 1: **Figure S1.** SAMHD1 T592A blocks LINE-1 replication in cycling cells. Same experiment as in Fig. 1, however, the mean of three independent experiments normalized on pcDNA transfected cells is shown. Error bars represent the standard deviation of the mean. Statistical analysis was performed using one way ANOVA followed by Tukey’s multiple comparison test. * *p* < 0.05; ** *p* < 0.01; *** *p* < 0.001; ns, not significant. One out of three independent experiments is shown. (PDF 16 kb)

Additional file 2: **Figure S2.** SAMHD1 T592A does not promote stress granule formation. (A) 293T cells were transfected with empty vector (pcDNA) or the non-phosphorylated SAMHD1-myc mutant T592A. Two days posttransfection, cells were probed with antibodies targeting the myc-tag (red) or endogenous G3BP1 as stress granule marker (cyan). As a positive control for stress granule formation, pcDNA-transfected cells were treated with 0.5 mM As_2_O_3_ for 1 h at 37 °C prior to fixation. Slides were analyzed by confocal microscopy. (B) 293T cells were transfected with the L1 expressions vector pAD2TE-O1F, encoding ORF1p-FLAG, or an expression vector for ORF1-FLAG alone together with empty vector (pcDNA) or SAMHD1-myc T592A. Two days posttransfection, cells were probed with antibodies targeting for ORF1-FLAG (green), SAMHD1-myc T592A (red), or the endogenous stress granule marker G3BP1 (cyan). Cells were analyzed by confocal microscopy. (C) A number of 100 cells for each transfection was examined to score stress granule (SG)-positive and –negative cells. The results are summarized in bar graphs. HeLa HA cells (D) or U2OS cells (E) were transfected with either an empty vector alone (pcDNA) or together with the constitutively active, non-phosphorylated SAMHD1 mutant (T592A). Two days posttransfection, cells were probed with anti-myc antibody targeting SAMHD1 T592A (red) or antibody targeting endogenous G3BP1 (cyan). Cells were analyzed by confocal microscopy. (PDF 203 kb)

## Abbreviations

AGS: Aicardi-Goutières syndrome
CDK: Cyclin-dependent kinase
dATP: Deoxyadenosine triphosphate
dNPT: Deoxynucleotide triphosphate
h: Hours
HIV: Human Immunodeficiency virus
HPLC: High-performance liquid chromatography
IAP: Intracisternal A-particle
L1: Long interspersed element 1
MLV: Murine leukemia virus
ORF1p: Open reading frame 1 protein
ORF2p: Open reading frame 2 protein
q: Quantitative
RNP: Ribonucleoprotein complex
RT: Reverse transcriptase
SAMHD1: SAM and HD domain-containing protein 1
SINE: Short interspersed element
T592: Threonine 592
TPRT: Target-primed reverse transcription
wt: Wild-type protein

## References

[1] Laguette N, Sobhian B, Casartelli N, Ringeard M, Chable-Bessia C, Segeral E, Yatim A, Emiliani S, Schwartz O, Benkirane M (2011). SAMHD1 is the dendritic- and myeloid-cell-specific HIV-1 restriction factor counteracted by Vpx. Nature.

[2] Hrecka K, Hao C, Gierszewska M, Swanson SK, Kesik-Brodacka M, Srivastava S, Florens L, Washburn MP, Skowronski J (2011). Vpx relieves inhibition of HIV-1 infection of macrophages mediated by the SAMHD1 protein. Nature.

[3] Baldauf HM, Pan X, Erikson E, Schmidt S, Daddacha W, Burggraf M, Schenkova K, Ambiel I, Wabnitz G, Gramberg T (2012). SAMHD1 restricts HIV-1 infection in resting CD4(+) T cells. Nat Med.

[4] Powell RD, Holland PJ, Hollis T, Perrino FW (2011). Aicardi-Goutieres syndrome gene and HIV-1 restriction factor SAMHD1 is a dGTP-regulated deoxynucleotide triphosphohydrolase. J Biol Chem.

[5] Goldstone DC, Ennis-Adeniran V, Hedden JJ, Groom HC, Rice GI, Christodoulou E, Walker PA, Kelly G, Haire LF, Yap MW (2011). HIV-1 restriction factor SAMHD1 is a deoxynucleoside triphosphate triphosphohydrolase. Nature.

[6] Franzolin E, Pontarin G, Rampazzo C, Miazzi C, Ferraro P, Palumbo E, Reichard P, Bianchi V (2013). The deoxynucleotide triphosphohydrolase SAMHD1 is a major regulator of DNA precursor pools in mammalian cells. Proc Natl Acad Sci U S A.

[7] Lahouassa H, Daddacha W, Hofmann H, Ayinde D, Logue EC, Dragin L, Bloch N, Maudet C, Bertrand M, Gramberg T (2012). SAMHD1 restricts the replication of human immunodeficiency virus type 1 by depleting the intracellular pool of deoxynucleoside triphosphates. Nat Immunol.

[8] Gramberg T, Kahle T, Bloch N, Wittmann S, Mullers E, Daddacha W, Hofmann H, Kim B, Lindemann D, Landau NR (2013). Restriction of diverse retroviruses by SAMHD1. Retrovirology.

[9] Tungler V, Staroske W, Kind B, Dobrick M, Kretschmer S, Schmidt F, Krug C, Lorenz M, Chara O, Schwille P, Lee-Kirsch MA (2013). Single-stranded nucleic acids promote SAMHD1 complex formation. J Mol Med (Berl).

[10] Goncalves A, Karayel E, Rice GI, Bennett KL, Crow YJ, Superti-Furga G, Burckstummer T (2012). SAMHD1 is a nucleic-acid binding protein that is mislocalized due to aicardi-goutieres syndrome-associated mutations. Hum Mutat.

[11] Beloglazova N, Flick R, Tchigvintsev A, Brown G, Popovic A, Nocek B, Yakunin AF (2013). Nuclease activity of the human SAMHD1 protein implicated in the Aicardi-Goutieres syndrome and HIV-1 restriction. J Biol Chem.

[12] Antonucci JM, St Gelais C, de Silva S, Yount JS, Tang C, Ji X, Shepard C, Xiong Y, Kim B, Wu L (2016). SAMHD1-mediated HIV-1 restriction in cells does not involve ribonuclease activity. Nat Med.

[13] Seamon KJ, Sun Z, Shlyakhtenko LS, Lyubchenko YL, Stivers JT. SAMHD1 is a single-stranded nucleic acid binding protein with no active site-associated nuclease activity. Nucleic Acids Res. 2015;43(13):6486–99. 10.1093/nar/gkv633 PMC4513882 26101257

[14] Ryoo J, Choi J, Oh C, Kim S, Seo M, Kim SY, Seo D, Kim J, White TE, Brandariz-Nunez A (2014). The ribonuclease activity of SAMHD1 is required for HIV-1 restriction. Nat Med.

[15] Choi J, Ryoo J, Oh C, Hwang S, Ahn K (2015). SAMHD1 specifically restricts retroviruses through its RNase activity. Retrovirology.

[16] Cribier A, Descours B, Valadao AL, Laguette N, Benkirane M (2013). Phosphorylation of SAMHD1 by cyclin A2/CDK1 regulates its restriction activity toward HIV-1. Cell Rep.

[17] White TE, Brandariz-Nunez A, Valle-Casuso JC, Amie S, Nguyen LA, Kim B, Tuzova M, Diaz-Griffero F (2013). The retroviral restriction ability of SAMHD1, but not its deoxynucleotide triphosphohydrolase activity, is regulated by phosphorylation. Cell Host Microbe.

[18] St Gelais C, de Silva S, Hach JC, White TE, Diaz-Griffero F, Yount JS, Wu L (2014). Identification of cellular proteins interacting with the retroviral restriction factor SAMHD1. J Virol.

[19] Pauls E, Ruiz A, Badia R, Permanyer M, Gubern A, Riveira-Munoz E, Torres-Torronteras J, Alvarez M, Mothe B, Brander C (2014). Cell cycle control and HIV-1 susceptibility are linked by CDK6-dependent CDK2 phosphorylation of SAMHD1 in myeloid and lymphoid cells. J Immunol.

[20] Yan J, Hao C, DeLucia M, Swanson S, Florens L, Washburn MP, Ahn J, Skowronski J (2015). CyclinA2-cyclin-dependent kinase regulates SAMHD1 protein Phosphohydrolase domain. J Biol Chem.

[21] Arnold LH, Groom HC, Kunzelmann S, Schwefel D, Caswell SJ, Ordonez P, Mann MC, Rueschenbaum S, Goldstone DC, Pennell S (2015). Phospho-dependent regulation of SAMHD1 oligomerisation couples catalysis and restriction. PLoS Pathog.

[22] Tang C, Ji X, Wu L, Xiong Y (2015). Impaired dNTPase activity of SAMHD1 by Phosphomimetic mutation of Thr-592. J Biol Chem.

[23] Rice GI, Bond J, Asipu A, Brunette RL, Manfield IW, Carr IM, Fuller JC, Jackson RM, Lamb T, Briggs TA (2009). Mutations involved in Aicardi-Goutieres syndrome implicate SAMHD1 as regulator of the innate immune response. Nat Genet.

[24] Zhao K, Du J, Han X, Goodier JL, Li P, Zhou X, Wei W, Evans SL, Li L, Zhang W (2013). Modulation of LINE-1 and Alu/SVA retrotransposition by Aicardi-Goutieres syndrome-related SAMHD1. Cell Rep.

[25] Hu S, Li J, Xu F, Mei S, Le Duff Y, Yin L, Pang X, Cen S, Jin Q, Liang C, Guo F (2015). SAMHD1 inhibits LINE-1 Retrotransposition by promoting stress granule formation. PLoS Genet.

[26] Lander ES, Linton LM, Birren B, Nusbaum C, Zody MC, Baldwin J, Devon K, Dewar K, Doyle M, FitzHugh W (2001). Initial sequencing and analysis of the human genome. Nature.

[27] Cordaux R, Batzer MA (2009). The impact of retrotransposons on human genome evolution. Nat Rev Genet.

[28] Denli AM, Narvaiza I, Kerman BE, Pena M, Benner C, Marchetto MC, Diedrich JK, Aslanian A, Ma J, Moresco JJ (2015). Primate-specific ORF0 contributes to retrotransposon-mediated diversity. Cell.

[29] Kazazian HH, Moran JV (2017). Mobile DNA in health and disease. N Engl J Med.

[30] Dewannieux M, Esnault C, Heidmann T (2003). LINE-mediated retrotransposition of marked Alu sequences. Nat Genet.

[31] Hancks DC, Kazazian HH (2016). Roles for retrotransposon insertions in human disease. Mob DNA.

[32] Moldovan JB, Moran JV (2015). The zinc-finger antiviral protein ZAP inhibits LINE and Alu Retrotransposition. PLoS Genet.

[33] Goodier JL, Pereira GC, Cheung LE, Rose RJ, Kazazian HH (2015). The broad-Spectrum antiviral protein ZAP restricts human Retrotransposition. PLoS Genet.

[34] Arjan-Odedra S, Swanson CM, Sherer NM, Wolinsky SM, Malim MH (2012). Endogenous MOV10 inhibits the retrotransposition of endogenous retroelements but not the replication of exogenous retroviruses. Retrovirology.

[35] Li X, Zhang J, Jia R, Cheng V, Xu X, Qiao W, Guo F, Liang C, Cen S (2013). The MOV10 helicase inhibits LINE-1 mobility. J Biol Chem.

[36] Goodier JL, Cheung LE, Kazazian HH (2012). MOV10 RNA helicase is a potent inhibitor of retrotransposition in cells. PLoS Genet.

[37] Kinomoto M, Kanno T, Shimura M, Ishizaka Y, Kojima A, Kurata T, Sata T, Tokunaga K (2007). All APOBEC3 family proteins differentially inhibit LINE-1 retrotransposition. Nucleic Acids Res.

[38] Athanasiadis A, Rich A, Maas S (2004). Widespread A-to-I RNA editing of Alu-containing mRNAs in the human transcriptome. PLoS Biol.

[39] Ostertag EM, Prak ET, DeBerardinis RJ, Moran JV, Kazazian HH (2000). Determination of L1 retrotransposition kinetics in cultured cells. Nucleic Acids Res.

[40] Dewannieux M, Dupressoir A, Harper F, Pierron G, Heidmann T (2004). Identification of autonomous IAP LTR retrotransposons mobile in mammalian cells. Nat Genet.

[41] Ribet D, Dewannieux M, Heidmann T (2004). An active murine transposon family pair: retrotransposition of "master" MusD copies and ETn trans-mobilization. Genome Res.

[42] Wittmann S, Behrendt R, Eissmann K, Volkmann B, Thomas D, Ebert T, Cribier A, Benkirane M, Hornung V, Bouzas NF, Gramberg T (2015). Phosphorylation of murine SAMHD1 regulates its antiretroviral activity. Retrovirology.

[43] Zhang R, Bloch N, Nguyen LA, Kim B, Landau NR (2014). SAMHD1 restricts HIV-1 replication and regulates interferon production in mouse myeloid cells. PLoS One.

[44] Bloch N, Glasker S, Sitaram P, Hofmann H, Shepard CN, Schultz ML, Kim B, Landau NR (2017). A highly active isoform of lentivirus restriction factor SAMHD1 in mouse. J Biol Chem.

[45] Yan J, Kaur S, DeLucia M, Hao C, Mehrens J, Wang C, Golczak M, Palczewski K, Gronenborn AM, Ahn J, Skowronski J (2013). Tetramerization of SAMHD1 is required for biological activity and inhibition of HIV infection. J Biol Chem.

[46] Kulpa DA, Moran JV (2006). Cis-preferential LINE-1 reverse transcriptase activity in ribonucleoprotein particles. Nat Struct Mol Biol.

[47] Faulkner GJ, Garcia-Perez JL. L1 mosaicism in mammals: extent, effects, and evolution. Trends Genet. 2017. 10.1016/j.tig.2017.07.004 28797643

[48] Lenzi GM, Domaoal RA, Kim DH, Schinazi RF, Kim B (2014). Kinetic variations between reverse transcriptases of viral protein X coding and noncoding lentiviruses. Retrovirology.

[49] Dai L, Huang Q, Boeke JD (2011). Effect of reverse transcriptase inhibitors on LINE-1 and Ty1 reverse transcriptase activities and on LINE-1 retrotransposition. BMC Biochem.

[50] Bhattacharya A, Wang Z, White T, Buffone C, Nguyen LA, Shepard CN, Kim B, Demeler B, Diaz-Griffero F, Ivanov DN (2016). Effects of T592 phosphomimetic mutations on tetramer stability and dNTPase activity of SAMHD1 can not explain the retroviral restriction defect. Sci Rep.

[51] Hulme AE, Bogerd HP, Cullen BR, Moran JV (2007). Selective inhibition of Alu retrotransposition by APOBEC3G. Gene.

[52] Doucet AJ, Hulme AE, Sahinovic E, Kulpa DA, Moldovan JB, Kopera HC, Athanikar JN, Hasnaoui M, Bucheton A, Moran JV, Gilbert N. Characterization of LINE-1 ribonucleoprotein particles. PLoS Genet. 2010;6(10). 10.1371/journal.pgen.1001150 PMC2951350 20949108

[53] Esnault C, Heidmann O, Delebecque F, Dewannieux M, Ribet D, Hance AJ, Heidmann T, Schwartz O. APOBEC3G cytidine deaminase inhibits retrotransposition of endogenous retroviruses. Nature. 2005;433:430–3. 10.1038/nature03238 15674295

[54] Kerns JA, Emerman M, Malik HS (2008). Positive selection and increased antiviral activity associated with the PARP-containing isoform of human zinc-finger antiviral protein. PLoS Genet.

[55] Thomas D, Herold N, Keppler OT, Geisslinger G, Ferreiros N (2015). Quantitation of endogenous nucleoside triphosphates and nucleosides in human cells by liquid chromatography tandem mass spectrometry. Anal Bioanal Chem.

[56] Diamond TL, Roshal M, Jamburuthugoda VK, Reynolds HM, Merriam AR, Lee KY, Balakrishnan M, Bambara RA, Planelles V, Dewhurst S, Kim B (2004). Macrophage tropism of HIV-1 depends on efficient cellular dNTP utilization by reverse transcriptase. J Biol Chem.

