# Correction to: The SAMHD1-mediated block of LINE-1 retroelements is regulated by phosphorylation

**DOI:** 10.1186/s13100-018-0121-8

**Published:** 2018-05-01

**Authors:** A. Herrmann, S. Wittmann, D. Thomas, C. N. Shepard, B. Kim, N. Ferreirós, T. Gramberg

**Affiliations:** 1 Institute of Clinical and Molecular Virology, Friedrich-Alexander University Erlangen-Nürnberg, Schlossgarten 4, 91054 Erlangen, Germany; 2 pharmazentrum frankfurt/ZAFES, Institute of Clinical Pharmacology, Goethe-University, Theodor Stern Kai 7, 60590 Frankfurt am Main, Germany; 3 Center for Drug Discovery, Department of Pediatrics, Emory Center for AIDS Research, Emory University, Children’s Healthcare of Atlanta, Atlanta, GA 30322 USA; 4 College of Pharmacy, Kyung-Hee University, Seoul, South Korea

## Correction

The original article [1] contains an omission in the Methods. The authors would like to note that the paragraph constituting the L1 promoter assay should read as the following:

L1 promoter assay

HEK-293T cells were transfected with the L1 promoter luciferase reporter plasmid (L1_RP_-luc) together with empty vector (pcDNA6mycHis) or SAMHD1 expressing vector using calcium phosphate. The reporter plasmid L1_RP_-luc and the L1 promoter activity assay have been described previously [2]. Two days posttransfection, cells were lysed with Cell Culture Lysis 5× reagent (Promega) and luciferase activity was quantified using commercially available components (Promega).
