# Supplementary figures and images for: The SAMHD1-mediated block of LINE-1 retroelements is regulated by phosphorylation
Source: Mob DNA. 2018 Mar 28;9:11. doi: 10.1186/s13100-018-0116-5 (PMC5872582; doi:10.1186/s13100-018-0116-5)

Figure S1

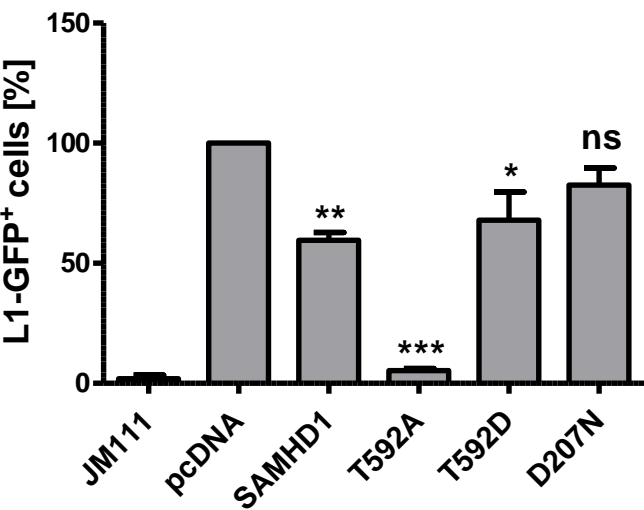

Supplement: Supplementary file 1 — Figure S1. SAMHD1 T592A blocks LINE-1 replication in cycling cells. Same experiment as in Fig. 1, however, the mean of three independent experiments normalized on pcDNA transfected cells is shown. Error bars represent the standard deviation of the mean. Statistical analysis was performed using one way ANOVA followed by Tukey’s multiple comparison test. * p < 0.05; ** p < 0.01; *** p < 0.001; ns, not significant. One out of three independent experiments is shown. (PDF 16 kb) [file 13100_2018_116_MOESM1_ESM.pdf]

# Figure S2

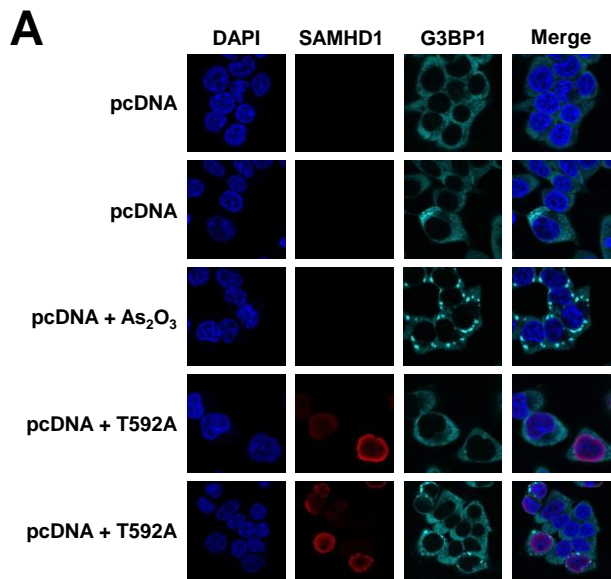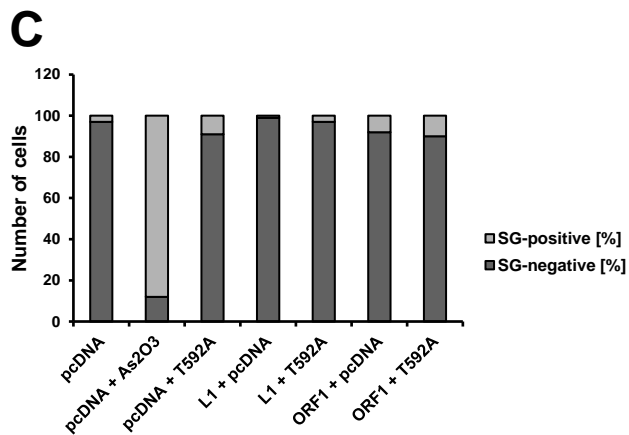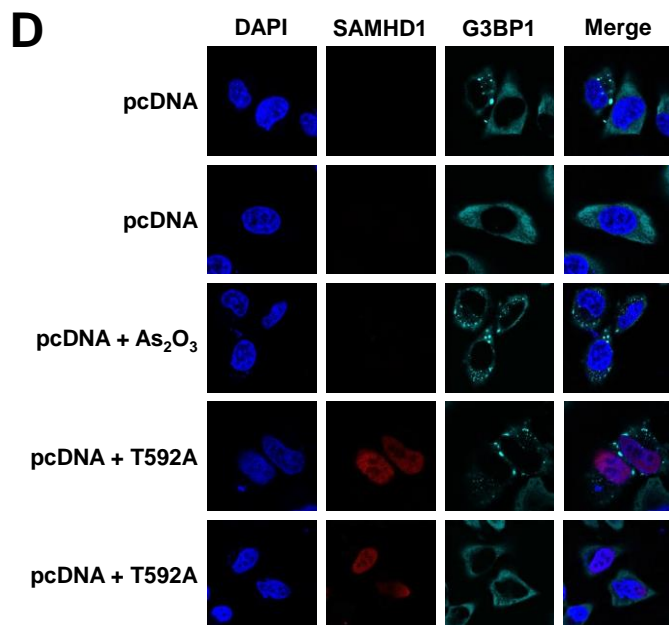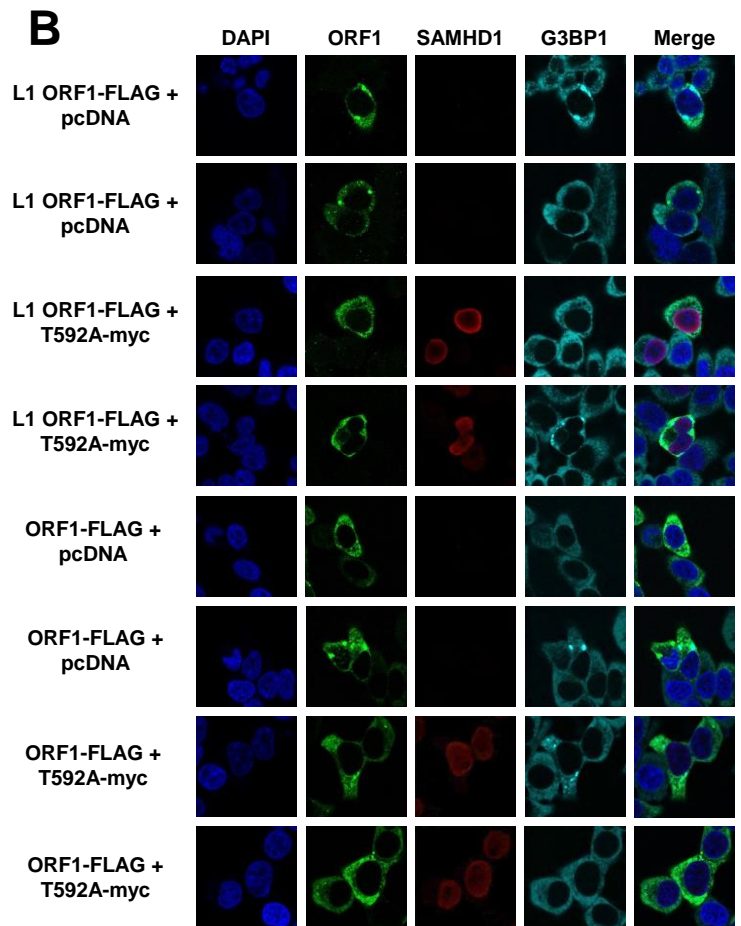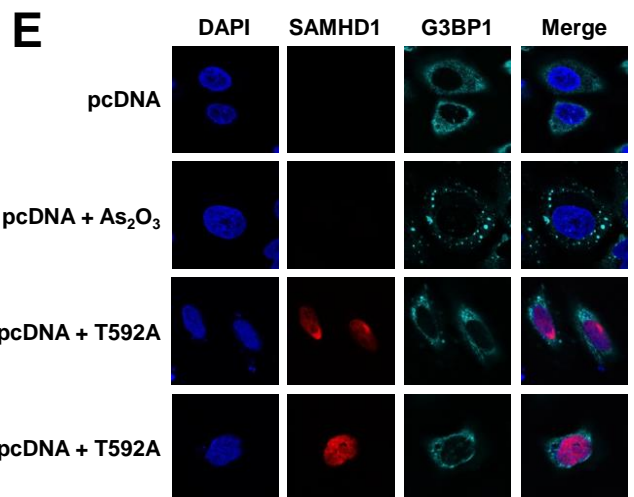

Supplement: Supplementary file 2 — Figure S2. SAMHD1 T592A does not promote stress granule formation. (A) 293T cells were transfected with empty vector (pcDNA) or the non-phosphorylated SAMHD1-myc mutant T592A. Two days posttransfection, cells were probed with antibodies targeting the myc-tag (red) or endogenous G3BP1 as stress granule marker (cyan). As a positive control for stress granule formation, pcDNA-transfected cells were treated with 0.5 mM As2O3 for 1 h at 37 °C prior to fixation. Slides were analyzed by confocal microscopy. (B) 293T cells were transfected with the L1 expressions vector pAD2TE-O1F, encoding ORF1p-FLAG, or an expression vector for ORF1-FLAG alone together with empty vector (pcDNA) or SAMHD1-myc T592A. Two days posttransfection, cells were probed with antibodies targeting for ORF1-FLAG (green), SAMHD1-myc T592A (red), or the endogenous stress granule marker G3BP1 (cyan). Cells were analyzed by confocal microscopy. (C) A number of 100 cells for each transfection was examined to score stress granule (SG)-positive and –negative cells. The results are summarized in bar graphs. HeLa HA cells (D) or U2OS cells (E) were transfected with either an empty vector alone (pcDNA) or together with the constitutively active, non-phosphorylated SAMHD1 mutant (T592A). Two days posttransfection, cells were probed with anti-myc antibody targeting SAMHD1 T592A (red) or antibody targeting endogenous G3BP1 (cyan). Cells were analyzed by confocal microscopy. (PDF 203 kb) [file 13100_2018_116_MOESM2_ESM.pdf]
